# Acceptance and compliance with micronutrient powder (MNP) among children aged 6–23 months in northern Nigeria

**DOI:** 10.1371/journal.pgph.0000961

**Published:** 2022-10-17

**Authors:** Stephen R. Kodish, Chris Isokpunwu, Tobi Osunkentan, Annette Imohe, Clara Ladi Ejembi, Stanley Chitekwe, Arjan de Wagt, Pragya Mathema

**Affiliations:** 1 Department of Nutritional Sciences, Pennsylvania State University, University Park, Pennsylvania, United States of America; 2 Department of Biobehavioral Health, Pennsylvania State University, University Park, Pennsylvania, United States of America; 3 Federal Ministry of Health, Abuja, Nigeria; 4 Nutrition Section, United Nations Children’s Fund (UNICEF), Abuja, Nigeria; 5 Ahmadu Bello University, Zaria, Nigeria; Medecins sans Frontieres, INDIA

## Abstract

This study sought to understand the utilization patterns and influencing factors of micronutrient powder (MNP) use among children aged 6–23 months in northern Nigeria as part of formative research to inform the design of an infant and young child feeding (IYCF) intervention. It had an iterative, multi-phase design whereby mixed methods data were collected from 144 households participating in an 8-week home-feeding trial. During the first four weeks, 12-hour direct observations were conducted with 24 households using MNP. Over the next four weeks, 18 of the same households were observed. In-depth interviews were also conducted among 27 caregivers to understand factors related to utilization. Unannounced spot checks (*n* = 86) were also conducted to gauge MNP compliance. Most households (76.7%) (66/86) adhered to instructions for using MNP (Adamawa (34/44 = 77.3%) and Kebbi (32/42 = 76.2%)). Facilitating factors to MNP adherence were identified, most notably the high ease of utilization, with 90.0% of caregivers indicating the MNP was ‘easy’ or ‘very easy’ to use. Several barriers to MNP compliance were identified and organized into three domains: product-related (e.g. difficulty opening sachet), child-related (e.g. not finishing fortified staple), and caregiver-related (e.g. difficulty making food daily). In Kebbi and Adamawa, MNP was accepted and utilized according to guidelines among most study participants. Findings may be used for scaling up MNP within a more comprehensive IYCF intervention in northern Nigeria.

## Introduction

Micronutrient deficiencies affect more than two billion people [1]. There is a disproportionate social and economic burden of vitamin and mineral deficiencies in low-income countries. To be sure, Nigeria loses $1.5 billion USD of gross domestic product per annum due to micronutrient deficiencies [2]. Many deficiency-related illnesses, including stunting, iodine deficiency, and iron-deficiency anemia, coupled with inadequate psychosocial stimulation, are key risk factors for compromised child development [3]. These nutrition-related challenges result in poor school enrolment and performance, decreased productivity and work capacity and consequently, reduced adult earning [3, 4]. Therefore, these issues warrant urgent attention for countries such as Nigeria where micronutrient deficiencies and less than optimal infant and young child feeding (IYCF) practices remain a public health problem.

In Nigeria, only 37% of children aged 6–23 months consume a minimally diverse diet (i.e., percentage of children 6–23 mo. who consumed foods and beverages from at least 5 of 8 defined food groups during the previous day) while just half (57%) are fed a minimum number of times (i.e., percentage of children 6–23 mo. who consumed solid, semi-solid, or soft foods at least 4 times during the previous day) [5–7]. Also, a minimally acceptable diet (i.e., percentage of children 6–23 mo. who consumed at least the minimum dietary diversity and minimum meal frequency for their age during the previous day) is consumed only by 18% of children 6–23 months while less than half (46%) consume iron-rich food during the previous day. Despite a child’s iron requirements being relatively higher between the ages of 6 and 11 months when growth, motor and cognitive development are the most rapid, the proportion of children who are fed foods rich in iron does increase with age in Nigeria but is the lowest among children ages 6 to 8 months and 9 to 11 months who happen to be the most in need [5, 6].

Sub-optimal IYCF practices are important nutrition challenges in northern Nigeria. For instance, in Adamawa, a northeastern state, only 14% of children aged 6–23 months has a minimally acceptable diet. And in Kebbi, a northwestern state, 22.6% of children of the same age group has such a diet [5]. Consequently, with 37% of children under-five suffering from stunting and 76.1% of pre-school aged children living with iron-deficiency anemia [8], these indicators of nutritional status in Nigeria reflect a nutrition situation that would benefit from intervention [6].

One viable intervention for improving upon nutritional status of a population is the home fortification of foods with powders containing multiple micronutrients. While other nutrition interventions (e.g., biofortification of staple crops) exist to address micronutrient deficiencies, home fortification has been recommended as one viable strategy to do so, especially iron-deficiency anemia, in children 6–23 months of age through consumption of one sachet per day [9]. In 2013, the Nigeria Federal Ministry of Health adopted this policy through review of the National Guidelines on Micronutrients Deficiencies Control to include the use of MNP for home fortification [10]. It is also endorsed in the current National Strategic Plan of Action for Nutrition (2014–2019) [11]. Evidence illustrates that use of MNP supplementation in young children can reduce anemia by up to 31% and iron deficiency anemia by up to 51% [12]. Despite this evidence, implementation of MNP programs is not without challenges around acceptability, coverage, adherence, and sustainability [13–15].

Interventions with specialized nutritious foods, including MNP, can face operational challenges when they use a superficial, top-down planning phase with little or no formative research [16]. Using formative research to gain an in-depth understanding of MNP acceptability and compliance among the target population in Adamawa and Kebbi is an imperative step for designing an effective IYCF-MNP intervention. This study therefore sought to describe household acceptance of and adherence to MNP, as well as to understand the primary barriers and facilitating factors to its adherence among children 6–23 months in Kebbi and Adamawa, Nigeria prior to program implementation.

## Methods

### Study setting

This study was conducted in Kebbi and Adamawa, two states of northern Nigeria. While the two sites share some characteristics, including the predominant religion of Islam, they are geographically distant (1,000 km apart) from one another with unique physical, social, and cultural environments that may differentially influence acceptability and compliance with MNP.

#### Kebbi state (northwest Nigeria)

Kebbi is in the northwestern geo-political zone of Nigeria, a landscape characterized by both flood plains of inland river valley systems and desert-like conditions that comprise one third of the state [17]. It has a population of 4.2 million and covers a total area of 36,800 km^2^ [18]. Agriculture, animal rearing and fishing are the major livelihoods. It is mainly populated by Hausa ethnicity. The prevalence of childhood stunting is 60.6%, which is the highest in Nigeria, and only 20% of households have access to improved sources of drinking water [6].

#### Adamawa state (northeast Nigeria)

Adamawa is in the northeastern zone of Nigeria, an area characterized by its mountains and valleys with short grasses and trees in the north compared to thicker vegetation in the south [19]. It has 4.0 million people and occupies a total area of 39,742 km^2^ making it one of the largest states in Nigeria [18]. The main livelihoods revolve around agriculture though fishing and cattle rearing are also common. While Hausa is spoken throughout the state, Adamawa is culturally diverse with more than fifty spoken languages and dialects. The childhood stunting prevalence is 34.3% [6].

### Study design, sampling, and data collection procedures

Data collection occurred in both states over three research phases and had an emergent design [20]. It was a descriptive, exploratory study that aimed to understand the local context for developing a culturally appropriate and scalable IYCF-MNP intervention in northern Nigeria. It drew from a Rapid Assessment Procedures (RAP) methodology designed for nutrition programs [21]. And it was modeled after similar formative nutrition research completed in Malawi and Mozambique [2, 22] with multiple, iterative phases. Data were collected by a team of ‘cultural insiders’ who were locally hired from Kebbi and Adamawa, or from similar cultural contexts in Nigeria [20]. They were trained for approximately 100 hours prior to fieldwork.

#### Phase 1: Exploring the sociocultural context for IYCF-MNP programming

In-depth interviews (*n* = 99) were conducted among community leaders such as village headmen (*n* = 18) and religious leaders (*n* = 15) (S1 File), as well as health workers (*n* = 30) (S2 File) and caregivers of children aged 6–23 months (*n* = 36) (S3 File) to understand the socio-cultural environment, including community perceptions toward nutrition-related illnesses and reported IYCF practices. This phase was also used to generate inputs for tailoring promotional materials (e.g., informative MNP brochure) that would accompany MNP distribution in phase 2 (S4 File).

#### Phase 2: Household feeding trial with MNP (weeks 1–4)

Phase 2 lasted 4 weeks during which 144 households (72 per state; 24 per child age range) participated in an eight-week, home-feeding trial with MNP. Households were eligible for participation if they met the following criteria: **1)** had a child aged 6–23 mo.; **2)** gave consent and child assent for full 8-week trial using MNP; and **3)** lived in an intervention area with no plan to move. Using these sampling criteria, the research team enrolled a total of 144 households in Kebbi (*n* = 72) and Adamawa (*n* = 72), with an equal number of children (*n* = 48) participating from each age stratum (6–11 mo., 12–17 mo., 18–23 mo.). At the start of the trial, each household received an 8-week supply of MNP (56 sachets).

Households were provided oral, as well as written and pictorial instructions (in the form of a tailored brochure developed by UNICEF on appropriate MNP usage, including several key messages: 1) Mix MNP into a small portion of semi-solid food and feed the fortified portion before feeding the remainder of the dish; 2) Provide one MNP sachet per child 6–23 months each day; 3) Provide MNP to that child for up to 60 days (i.e., through 8 weeks feeding trial duration); 4) In the case that a day or more of MNP provision is missed, then still continue to follow the one MNP sachet per child each day instructions thereafter; 5) Do not add MNP to food during cooking; and 6) Do not add MNP to water or other liquids (S4 File). The content of the brochure was informed in part by phase 1 interview findings reflecting design preferences. Oral instructions were also provided in Hausa or Chamba by community health workers familiar to the caregivers at time of distribution.

All 144 participating households participated in at least one of three data collection methods (full-day observations, in-depth interviews, spot checks) during the eight weeks. Given the capacity of the research team and fieldwork conditions at the time, as well as in consideration of participant burden, data were collected from each household using just one, and in some cases two, methods. For instance, a household that participated in an in-depth interview was neither spot checked nor observed, while another household that participated in a full-day observation was not interviewed. During the eight weeks, nearly all 144 households were visited at least one time for data collection. No households participated in all three methods of data collection.

Phase 2 included the first round of full-day (12-hour) observations of 24 households (12 per state) (S5 File). Households were purposively sampled firstly based on child age to ensure 8 children per age stratum (6–11 mo., 12–17 mo., 18–23 mo.). Additional criteria were then applied to the sampling strategy in consideration of similar geographic representation by state, LGA, and community units. The same households participating during this first round were also observed approximately four weeks later during phase 3 to reduce possible reactivity associated with being observed. Sample sizes were determined based on both feasibility and previous research using direct observations to understand utilization of specialized nutritious foods [16, 22, 23].

#### Phase 3: Household feeding trial with MNP (weeks 5–8)

Phase 3 also lasted 4 weeks and included the second round of full-day observations among 18 of the 24 participating phase 2 households. Six of the original 24 households were not observed a second time due to unavoidable fieldwork challenges in Kebbi at that time.

To triangulate observational data, we conducted 27 in-depth interviews (14 in Kebbi; 13 in Adamawa) to understand caregiver experiences using MNP. Interviews were conducted in Hausa using a semi-structured guide and digitally recorded for translation into English (S6 File). Data were collected until saturation was reached among key themes [24].

Finally, unannounced spot checks were conducted among 86 households to gauge MNP compliance throughout the eight weeks. Spot checks are an observational method that we used in this study to record the number of MNP sachets remaining in each household for comparison to the number of sachets that should remain based on the date of MNP distribution at the start of the trial. Open-ended questions related to ease or difficulty of MNP compliance were posed to caregivers during each spot check and recorded as field notes on a semi-structured form (S7 File). Households were purposively sampled for spot checks also in consideration of geographic representation (by state and LGA) and child age. Sample sizes were determined based on feasibility as well as previous research utilizing spot checks to understand household compliance with similar specialized nutritious foods [25].

### Data analysis

#### In-depth interviews

Textual data from interviews were translated and transcribed from Hausa and Chamba into English. The team transcribed data from the digital recordings verbatim, maintaining *emic* terminology to minimize losing valuable meaning in local phrases. Dedoose software was used for data management and coding [26]. Data were coded shortly after transcriptions became available using unique codebooks for each type of participant.

Within Dedoose, the textual data were analyzed drawing from aspects of Grounded Theory [27]. The process began with broad coding until salient themes were identified. Then focused coding was applied across transcripts to identify sub-themes. During the coding process, analytic notes (i.e., memos) were made within Dedoose to make constant comparisons between and among themes and sub-themes [27]. Finally, interpretation of the coded text was made based on the research questions and in consideration of program design.

#### Direct observations

Direct observations yielded both qualitative (e.g. descriptive field notes related to food preparation and feeding behaviors) and quantitative (e.g. frequencies of giving or receiving food, episodes of reactivity, etc.) data. Observers recorded behaviors based on events of interest related to MNP utilization, as well as based on time: descriptions of young child activities were recorded at minimum every 5 minutes [28]. Both textual and numerical data from direct observation forms were aggregated across observations and then stratified by geographic units and age group of index child (6–11 mo.; 12–17 mo.; 18–23 mo.).

#### Spot checks

Spot checks of household MNP stocks were conducted to gauge compliance with the recommended dose of one sachet per child per day. To do so we created three categorical definitions **(strict:** ±0 MNP sachets; **flexible**: ±3 sachets; and **low**: >±4 sachets room for error) based on the number of MNP sachets remaining in a household compared to the number that should have been remaining based on the specific date of MNP distribution. For example, a household with 14 sachets remaining after 6 weeks (i.e., 42 sachets used over 42 days) was classified as a “strict” user, whereas had 17 sachets remained then it was considered to be a “flexible” user. We defined a “compliant user” as the total number of strict and flexible users. Findings are presented by state, LGA, and index child age.

#### Ethical approval

The study protocol was approved by the National Health Research Ethics Committee of Nigeria. All adult participants provided verbal informed consent witnessed by a field supervisor. Parents, or legal guardians, also provided verbal assent for their children prior to participate in the study.

## Results

### Compliance with MNP

Overall, 76.7% (66/86) of households either strictly or flexibly used the MNP, with no substantial difference between Adamawa (34/44 = 77.3%) and Kebbi (32/42 = 76.2%). Based on our definitions, 23.2% of households (20/86) had a low adherence, with similar percentages between Adamawa (10/44 = 22.7%) and Kebbi (10/42 = 23.8%) (Table 1).

**Table 1:** Spot check data by type of user and geographic location.

Location	Strict Compliance	Flexible Compliance	Compliant Users	Low Compliance
Song	0/13 (0.0%)	13/13 (100.0%)	13/13 (100.0%)	0/13 (0.0%)
Ganye	2/18 (11.1%)	12/18 (66.7%)	14/18 (77.8%)	4/18 (22.2%)
Guyuk	1/13 (7.7%)	6/13 (46.2%)	7/13 (53.8%)	6/13(46.2%)
**Adamawa**	3/44 (6.8%)	31/44 (70.5%)	34/44 (77.3%)	10/44 (22.7%)
Birnin Kebbi	1/16 (6.3%)	11/16 (68.8%)	12/16 (75%)	4/16 (25.0%)
Jega	3/11 (27.3%)	8/11 (72.7%)	11/11 (100)	0/11 (0.0%)
Danko-wasagu	3/15 (20.0%)	6/15 (40.0%)	9/15 (60.0%)	6/15 (40.0%)
**Kebbi**	7/42 (16.7%)	25/42 (59.5%)	32/42 (76.2%)	10/42 (23.8%)
**Total**	10/86 (11.6%)	56/86 (65.1%)	66/86 (76.7%)	20/86 (23.2%)

By using a **strict definition of adherence** (±0 MNP sachets room for error), spot check data indicate that only 6.8% (3/44) and 16.7% (7/42) households in Adamawa and Kebbi, respectively, complied with MNP usage. No substantial differences emerged across age groups of children or by LGA level. Spot check findings using a strict definition of adherence and stratified by LGA and index child age range are presented in Table 2.

**Table 2:** Strict MNP users (±0) according to spot check data by age group and location.

Location	6–11 months	12–17 months	18–23 months	6–23 months
Song	0/3 (0.0%)	0/7 (0.0%)	0/3 (0.0%)	0/13 (0.0%)
Ganye	2/7 (28.6%)	0/6 (0.0%)	0/5 (0.0%)	2/18 (11.1%)
Guyuk	1/4 (25.0%)	0/1 (0.0%)	0/8 (0.0%)	1/13 (7.7%)
**Adamawa**	3/14 (21.4%)	0/14 (0.0%)	0/16 (0.0%)	3/44 (6.8%)
Birnin Kebbi	0/7 (0.0%)	1/6 (16.7%)	0/3 (0.0%)	1/16 (6.3%)
Jega	1/4 (25.0%)	1/3 (33.3%)	1/4 (25.0%)	3/11 (27.3%)
Danko-wasagu	1/4 (25.0%)	1/7 (14.3%)	1/4 (25.0%)	3/15 (20.0%)
**Kebbi**	2/15 (13.3%)	3/16 (18.8%)	2/11 (18.2%)	7/42 (16.7%)

By using a **flexible definition of adherence** (±3 sachets room for error) the percentage of participants with an appropriate supply of MNP remaining based on the distribution cycle increased to 70.5% (31/44) and 59.5% (25/42) of households in Adamawa and Kebbi, respectively. The LGA with the highest adherence based on this definition was Song, Adamawa where 100.0% of households (13/13) had an appropriate supply of MNP at time of spot check. The LGA with the lowest adherence was Danko-Wasagu, Kebbi where 40.0% of households (6/15) had an appropriate supply. The spot check findings using a flexible definition of adherence and stratified by LGA and index child age range are presented in Table 3.

**Table 3:** Flexible MNP users (±3) according to spot check data by age group and location.

Location	6–11 months	12–17 months	18–23 months	6–23 months
Song	3/3 (100.0%)	7/7 (100.0%)	3/3 (100.0%)	13/13 (100.0%)
Ganye	4/7 (57.1%)	4/6 (66.7%)	4/5 (80.0%)	12/16 (75.0%)
Guyuk	0/4 (0.0%)	0/1 (0.0%)	6/8 (75.0%)	6/13 (46.2%)
**Adamawa**	7/14 (50.0%)	11/14 (78.6%)	13/16 (81.3%)	31/44 (70.5%)
Birnin Kebbi	5/7 (71.4%)	4/6 (66.7%)	2/3 (66.7%)	11/16 (68.8%)
Jega	3/4 (75.0%)	2/3 (66.7%)	3/4 (75.0%)	8/11 (72.7%)
Danko-wasagu	1/4 (25.0%)	2/7 (28.6%)	3/4 (75.0%)	6/15 (40.0%)
**Kebbi**	9/15 (60.0%)	8/16 (50.0%)	8/11 (72.7%)	25/42 (59.5%)

By summing the strict and flexible MNP users we determined that 76.7% (66/86) of households had an appropriate supply of MNP remaining. Based on this figure and considering our **definition of low-adherence** (>±4 sachets room for error), we calculated the percentage of low-adherence users (Table 4). We determined that 23.3% (20/86) of households did not have an appropriate household MNP supply at the time of spot check.

**Table 4:** Low-compliance MNP users (>±4) according to spot check data by age group and location.

Location	6–11 months	12–17 months	18–23 months	6–23 months
Song	0/3 (0.0%)	0/7 (0.0%)	0/3 (0.0%)	0/13 (0.0%)
Ganye	1/7 (14.3%)	2/6 (33.3%)	1/5 (20.0%)	4/18 (22.2%)
Guyuk	3/4 (75.0%)	1/1 (100.0%)	2/8 (25.0%)	6/13 (46.2%)
**Adamawa**	4/14 (28.6%)	3/14 (21.4%)	3/16 (18.8%)	10/44 (22.7%)
Birnin Kebbi	2/7 (28.6%)	1/6 (16.7%)	1/3 (33.3%)	4/16 (25.0%)
Jega	0/4 (0.0%)	0/3 (0.0%)	0/4 (0.0%)	0/11 (0.0%)
Danko-wasagu	2/4 (50.0%)	4/7 (57.1%)	0/4 (0.0%)	6/15 (40.0%)
**Kebbi**	4/15 (26.7%)	5/16 (31.3%)	1/11 (9.1%)	10/42 (23.8%)

### MNP utilization during feeding

Caregivers in Adamawa and Kebbi used similar feeding practices when fortifying the MNP into complementary foods. Largely, the MNP was fortified into typical young child porridges in line with the instructions provided at the beginning of the home-feeding trial.

In Adamawa, observations suggest MNP was utilized appropriately during most feeding episodes. Out of 24 observations, 87.5% (21/24) of children were fed fortified porridge with MNP (Table 5). Typical household usage involved appropriate MNP adherence by fortification into small portions of semi-solid foods including *kunu* varieties such as *dawa* and *akamu*, without any observed sharing. Data indicated that children’s *kunu* was not fortified with soy flour or other local foods before, during, or after meal preparation. We found that young children aged 6–11 months are uniquely favored during meals compared to their older siblings: they are mainly breast fed, given special attention by caregivers, sometimes are given special meals such as powdered milk, biscuits (cookies). We observed no mothers washing their children’s hands before feeding them but most did so after meals.

**Table 5:** Household MNP adherence in Kebbi and Adamawa among children 6–23 months.

	Kebbi (*n* = 18)	Adamawa (*n* = 24)
**Was MNP used during the 12-hour observation?**	17/18 (94.4%)	21/24 (87.5%)
**Was MNP given to index child *only* once during day?**	15/17 (88.2%)	21/21 (100%)
**Was MNP added to a semi-solid food *after* cooking?**	14/17 (82.4%)	21/21 (100%)
**Was MNP consumed *only* by the beneficiary child?**	11/16 (68.8%)	21/21 (100%)

*Data derived from direct household observations;

**During some observations the specific behavior(s) in question could not be assessed, thus different denominators appear above

**In Kebbi,** >75% of the direct observations indicated MNP compliance. Those households that did not appropriately utilize MNP did so in the form of sharing or adding MNP to a quantity of food that the child could not finish. This practice resulted in some children consuming less than one MNP sachet per day due to leftovers. Also, some mothers used cold, watery porridge (*pap*) into which they sometimes mixed MNP. Also, children aged 6–11 months are unique in that they are mainly breastfed and favored compared to other household children. Caregivers primarily added MNP to varieties of *kunu* (e.g., akamu and *koko*). Uniquely in Kebbi, there was a wide variety of foods that caregivers used for fortification, including types of thickened soups, *shinkafa*, *tuwo*, *fura*, and *miya*. Only 5% (2/42) of households practiced handwashing prior to child feeding.

### Facilitating factors of MNP compliance

**Fig 1:**
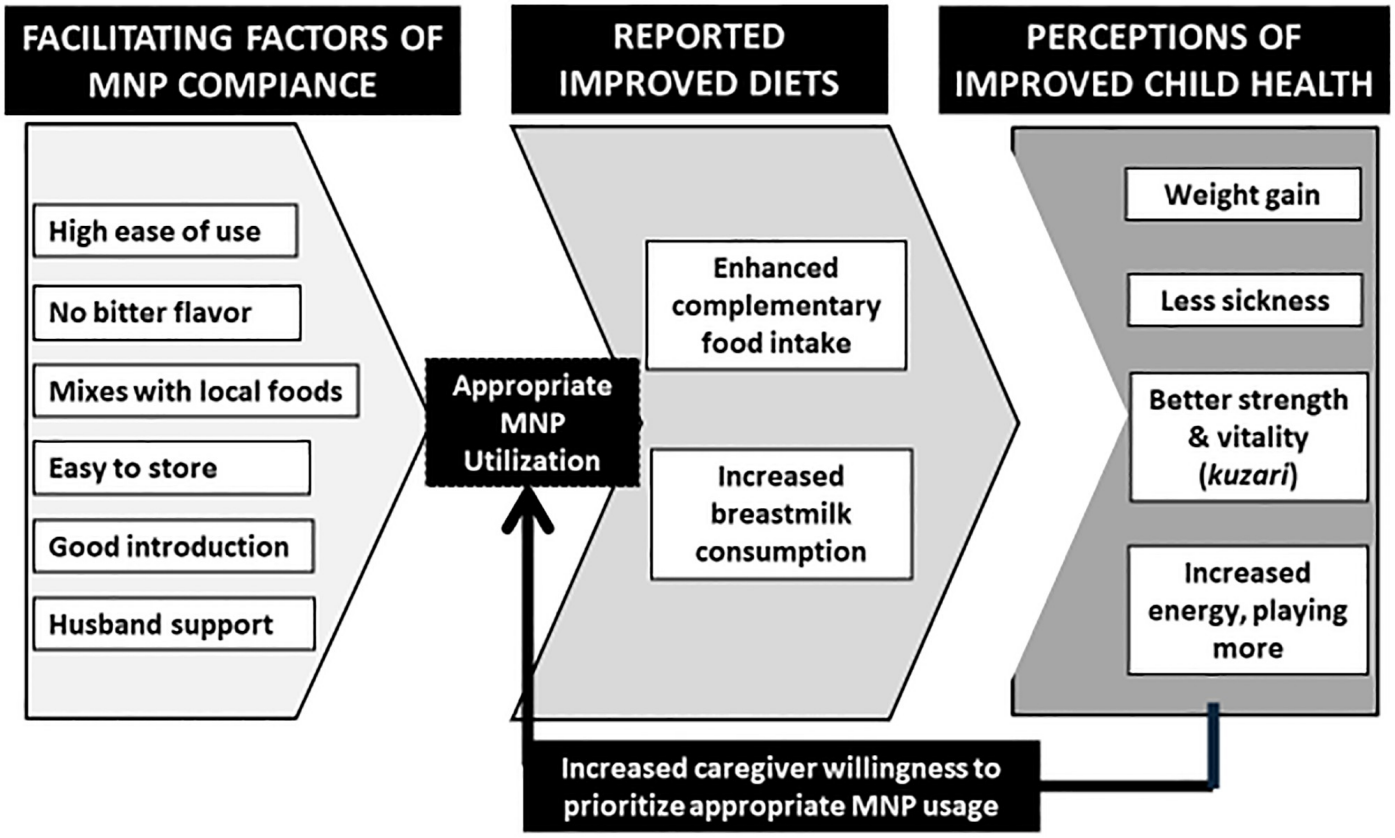
Facilitating factors of micronutrient powder (MNP) compliance. Six facilitating factors emerged from spot check and interview data related to MNP acceptance and compliance: high ease of use, “no bitter flavor,” mixes with local foods, easy to store MNP,” good introduction,” and husband support.

There were three types of benefits that contributed to positive caregiver perceptions: 1) facilitating factors to MNP acceptance and utilization; 2) improved infant and young child feeding practices; and, 3) perceived improved health of children 6–23 months after MNP usage. Data suggest that these factors, synergistically, formed a positive behavioral feedback loop to appropriate MNP utilization (Fig 1).

#### High ease of use

Overall, 88.8% (71/80) of caregivers that consented to a spot check indicated that the MNP was “*easy*” or “*very easy*” to use regularly (Table 6).

**Table 6:** Kebbi and Adamawa spot check data indicating perceived ease of MNP usage.

State	Very Difficult	Difficult	Easy	Very Easy
Adamawa (*n* = 42)	1	7	18	16
Kebbi (*n* = 38)	0	1	15	22
Total*	1	8	33	38
	9/80 (11.3%)		71/80 (88.8%)	

*4 households did not give consent during spot check data collection

Interview data corroborate this finding. Caregivers stressed that the MNP was appropriate for both themselves and their children because it was “*easy to use*”. A caregiver from Adamawa pointed out that when she was introduced to MNP, the health worker provided a demonstration to illustrate how to use it. She said, *“I have seen the use of the product and that is what makes it easy to use MNP*.*”*

#### No bitter flavor

Most caregivers indicated that the MNP was tasteless with “*no bitter flavor*” and a primary reason why caregivers indicated the product to be ‘*easy’* or ‘very easy’ to use. Some caregivers indicated that their children would refuse the fortified food (i.e., the staple food vehicle) if they saw the MNP being added to it; other caregivers explained that their children liked to see the MNP being added, as it was perceived similarly to sugar or other tasty food additives. Overall, children did not have reactions to the flavor of the MNP and refusals to eat were from a rejection of the staple food, not the MNP, according to caregivers. Also, caregivers indicated that unlike other medicines with which they are familiar, in fact the MNP had “*no bitter flavor*” so children were able to consume it without complaint. A caregiver in Adamawa said, “*The fact that the product does not taste bitter is very satisfactory to me because our children usually reject anything that is bitter*.”

#### Mixes with local foods

Caregivers explained that MNP was easily added to local foods in line with existing IYCF practices. Locally, in Kebbi and Adamawa, there are common young child foods that are semi-solid so caregivers did not have to make major changes to existing IYCF practices to use the MNP appropriately.

“*The MNP is easy to use because it doesn’t involve the swallowing of a tablet…just to be mixed with common food that is easily accessible and easy to prepare*. *It mixes well with the kunu*, *and its presence in kunu can hardly be detected*. *So children simply consume the MNP*…*without detecting its presence in the meal*.”

-Interview with female caregiver, Adamawa, Nigeria

*Kunu* was the main food used during home fortification, but it was also often prepared using a very watery consistency, making the MNP sometimes difficult to mix.

#### Easy to store

To a lesser extent, caregivers indicated that the MNP was convenient to store safely. A caregiver from Bernin Kebbi, Kebbi noted, **“***It was easy to store it because of the size***.”** Most people indicated that storage “*was not a problem*”. Caregivers explained easy storage on the top of “*cupboard*,” “*bookshelf*,” or “*in a box with books*,” “*a drawer*,” or “*traditional pot with cover*.”

#### Good introduction

The *“good introduction*” of the MNP to households during the home-feeding trial was explained to be a facilitating factor for household acceptance and compliance. The MNP was distributed to households only after advocacy meetings and courtesy calls at state and local government levels. The MNP was then distributed by recognized community health workers familiar to community members. This process was important for MNP acceptance and adherence, according to interview participants.

#### Husband support

Some caregivers who used the MNP in the trial explained that they received support from their husbands in the form of reminders and encouragement. There were two households, however, where husbands did not allow female caregivers to use MNP.

#### Reported improved diets

Caregivers reported that their children had increased appetites after consuming MNP throughout the trial period.

“*Since I started giving her MNP I noticed that she has added weight and has a better appetite*. *She eats more than she did before*. *I also noticed that she is healthier and has not had any illness since then*. *Breastfeeding has increased also and she has so much energy*.”

**-**Interview with female caregiver, Adamawa, Nigeria

Overall, caregivers reported more willingness among children to eat more foods during meal and non-meal times (e.g. in the middle of the night). One mother in Adamawa explained that “*previously my child ate 1 cup cerelac*, *but now 1*.*5 to 2 cups*” and another said, “*…before she only ate 4 or 5 spoonfuls*, *but now she eats more food*”. Several caregivers in Kebbi indicated that there were noticeable improvements in the quantity of *tuwo* consumed by children. Some caregivers suggested that as food intake increased, breastfeeding decreased though.

#### Perceptions of improved child health

Four primary themes emerged in relation to the caregiver perceptions that MNP improved the health status of their children during the trial: perceived weight gain, fewer bouts of illness, strength and vitality (*kuzari*), and increased energy, playing more (less lethargy).

*Weight gain*. Caregivers reported positive health effects, such as weight gain, from their children’s MNP consumption.

“*The MNP has improved my child’s health and weight*. *Before introducing MNP to us*, *she was weighing 6*.*4 to 7*.*4 kilograms but last Wednesday she was weighing up to 9*.*6 kilograms*. *Since I have seen how MNP increases my child’s health and weight*, *I believe it’s good for children*.”

-Interview with female caregiver, Adamawa, Nigeria

*Less sickness*. The second-most salient theme that emerged in relation to MNP impact on child health was “*less sickness*” such as anemia.

“*My first child suffers from rashin jiki (anemia) and we went to the clinic for a medical check-up and the doctor told us that she is suffering from rashin jiki*…*two months later*, *you see the way she is playing*, *running up and down…you get to know that she is now stronger and healthier*…”

-Interview with female caregiver, Kebbi, Nigeria

Caregivers also referred to improvements in their children’s complexion, saying their children “*look good*” or are *“looking fresh*.” Caregivers also explained that that they believed MNP was a form of protection from illness.

*Better strength & vitality (kuzari)*. Caregivers emphasized that MNP made their children “*stronger*” and “*have increased strength and vitality*.”

“*If you have two children*…*one administered MNP and the other not administered it*, *then you will surely see the clear difference*…*it may be in the kuzari* [vitality] *of their body*, *health*, *and strength…the one not using MNP will look weaker than*, *and not as healthy as the other one using it*.”

-Interview with female caregiver, Kebbi, Nigeria

Caregivers in both states placed high value on a child that has *kuzari*. Similarly, they emphasized the importance of, “*MNP is a body-building product*,” referring to increased child strength.

*Increased energy*, *playing more (less lethargy)*. MNP also reportedly increased child activity levels. A female caregiver from Adamawa explained, “*Now that we are using MNP the child dances*, *plays with his brother*, *and does whatever he wants to do*.” These facilitating factors synergistically contributed to MNP adherence: caregivers reported improved IYCF practices that led to perceptions of child health improvements, contributing to a positive behavioral feedback loop.

### Barriers to MNP compliance

Barriers to appropriate MNP adherence were also identified through interviews. The barriers could be categorized into three categories: product-related, child-related, and caregiver-related factors of MNP acceptance and compliance.

#### Product-related factors

*Difficulty opening the MNP sachet*. Caregivers explained that they often had to use their teeth, knife, or blade to open the MNP sachet before fortifying their children’s food.

“*I would not say it is a challenge but unsealing the sachet is somehow difficult for me*. *Some days I use my teeth and the powder may pour on the ground*.”

-Interview with female caregiver, Adamawa, Nigeria

*Clumping of MNP*. *S*ome participants reported the MNP would “*clump”* or “*not mix well”* into local foods.

“*The only challenge is that the product does not dissolve quickly and when poured into ‘kunu’ it creates lumps until I mix it for three or four minutes*.”

-Interview with female caregiver, Adamawa, Nigeria

#### Child-related factors

*Not eating the MNP staple food vehicle*. Most commonly, caregivers explained that they had trouble getting their children to consume MNP because of staple food refusals.

“*Some days my child rejects the kunu mixed with MNP so what I do is force her to eat it*. *That is the only difficulty I have encountered*.”

-Interview with female caregiver, Adamawa, Nigeria

Caregivers explained resorting to traditional forced feeding practices in many cases.

*MNP side effects*. Caregivers in Adamawa, and to a lesser extent, Kebbi, reported that their children had initial gastrointestinal troubles from MNP, with diarrhea as the primary manifestation. These caregivers either stopped using MNP or saw a pharmacist for treatment. Several caregivers indicated that they were encouraged by their husbands, other family members, or program staff to continue using MNP despite the diarrhea.

#### Caregiver-related factors

*Commitment*. Finally, caregivers explained home fortification to be a commitment, given other household demands. This commitment entailed remembering to use it, as well as daily preparation of a semi-solid food for fortification. Data suggest that caregivers typically preferred preparing cereal-based porridges one or several nights before each feeding episode so having to prepare a semi-solid porridge everyday was not aligned with typical food preparation practices in this context.

## Discussion

This formative research, which included an eight-week usability trial with 144 households, suggests that MNP is likely to be accepted and well utilized in Kebbi and Adamawa states as part of a larger IYCF-MNP intervention.

The spot checks and direct observations yielded good acceptability and adherence to the MNP, and aligned with similar MNP adherence in other studies in Nigeria [15]. We first attribute the findings to careful MNP distribution using a community-based model that engaged community health workers familiar to the households. An MNP-IYCF intervention in Madagascar also found a community-based model to be effective [29]. Second, we ascribe them to the phased formative research design, which allowed us to incorporate Phase 1 findings (e.g., identification of emic Hausa terms, such as *kuzari*, to tailor MNP-related messaging) into the usability trial where MNP was introduced. Third, through a literature review, we could communicate the expected benefits and potential side effects of MNP to caregivers during distribution, thus helping to alleviate negative rumor generation. Ample global evidence now reports similar factors across MNP interventions and thus can be used in pre-emptive messaging efforts to improve adherence [14, 16, 29–31].

This work revealed a variety of factors that contributed to MNP acceptance and adherence. Caregiver perceptions of the MNP itself (e.g., “high ease of use”, “no bitter flavor”, “mixes with local food”, “easy to store”) and the associated health benefits for children were salient themes in this study. And just as household support (e.g., “husband support”) proved important for adherence so too did “good introduction” by community health workers during MNP distribution at the beginning of the eight-week trial. Most studies of MNP acceptability and adherence have revealed similar types of factors across diverse contexts, thus highlighting important domains of inquiry during formative phases of MNP-related programming [14, 16, 29, 31–35]. Understanding the range of context-specific factors that influences individual acceptability and compliance with specialized nutritious foods may help inform tailored programming.

The implications of this research went beyond informing the scale up of MNP-IYCF programming in northern Nigeria. Findings were also used to develop SBCC materials to introduce MNP as part of a larger nutrition response in Borno state where insecurity has caused displacement and food insecurity since 2015. A primary critique of formative research is that it is context-specific and thus unable to be used outside of the context where it was conducted. However, this study is one example of effectively using formative findings for a related, yet unplanned nutrition action. Making unpublished reports available more widely is one way to facilitate this type of transferability and should be an imperative of knowledge management efforts within organizations.

This study had several strengths. First, the emergent and iterative nature of this work enabled us to incorporate phase 1 findings into the home-feeding trial. This design likely enhanced MNP acceptance and adherence as we overcame potential socio-cultural and service delivery barriers prior to its introduction. Second, we used methodological triangulation to understand MNP adherence through multiple methods. Doing so gives us confidence in the credibility of our findings and provides different perspectives from which to understand MNP adherence. Third, this usability trial was eight weeks. While this duration was not long enough to assess “continued use” of MNP after two months, we were able to assess adherence considering both“initiation” and“appropriate use” during this time [14].

The research also had limitations. We collected MNP adherence data over two months so season-specific findings are likely. Data about MNP adherence through program monitoring should inform implementation across seasons. Finally, reactive behaviors of household members during direct observations likely occurred–that is, participants may have changed their own behaviors while being observed. We used repeated observations of the same 24 households to reduce this phenomenon. While there is evidence to suggest that reactive meal behaviors decrease substantially after the first home observation [36], there is a research need to reaffirm this finding across contexts, especially where specialized nutritious foods are being introduced within humanitarian programs.

## Conclusions

This study highlights the utility of systematic formative research for developing an IYCF-MNP intervention requiring social and behavior change. It also highlights the continued commitment of the Nigeria government to address malnutrition and work toward Sustainable Development Goal 2 [*End hunger*, *achieve food security*, *and improved nutrition and promote sustainable agriculture*] using evidence-based approaches [37].

While findings from this usability trial were positive, addressing factors at multiple behavioral levels through tailored programming remains critical for creating an enabling environment where caregivers can improve the nutrition of young children during IYCF-MNP program scale up.

## Supporting information

S1 File Community leader interview guide (phase 1). (PDF)

S2 File Health worker interview guide (phase 1). (PDF)

S3 File Caregiver of children 6–23 months interview guide (phase 1). (PDF)

S4 File Tailored brochure distributed to households in MNP usability trial (phase 2). (PDF)

S5 File Abbreviated full-day, direct observation form (phases 2–3). (PDF)

S6 File Caregivers in MNP usability trial interview guide (phase 3). (PDF)

S7 File Spot check data collection instrument (phase 3). (PDF)

S1 Dataset Sources of data underlying key findings. (ZIP)
