# Supplementary material for: Acceptance and compliance with micronutrient powder (MNP) among children aged 6–23 months in northern Nigeria
Source: PLOS Glob Public Health. 2022 Oct 17;2(10):e0000961. doi: 10.1371/journal.pgph.0000961 (PMC10022258; doi:10.1371/journal.pgph.0000961)
Supplement: S1 File — (PDF) [file pgph.0000961.s001.pdf]

COMMUNITY LEADER INTERVIEW GUIDE
(ENGLISH VERSION)

Introduction

- **Thank you for speaking to me today. Please start by telling me a little bit about your community.**
- **Describe for me your role as the community leader please.**
- **Can you please discuss the challenges that your community faces?**
  - Probe on food-related challenges

Community Overview

- **Tell me about the dynamics of community life here**
  - Probe on ethnic group, language, and kinship
  - Probe on gender roles
  - Probe on leadership hierarchy
  - Probe on traditional healers in the community
  - Probe on what defines a household in this setting

Illnesses in the Community

- **You mentioned some different challenges. Can you tell me more about the illnesses that your community suffers from?**
  - Probe on how they are caused
  - Probe on their seriousness
  - Probe on ways to prevent them

- **Now could you talk about the nutrition or food-related illnesses in your community?**
  - Probe on whether micronutrient deficiency (mention symptom) is an illness
  - Probe on whether being too short (stunting) is an illness

- **I am most interested in illnesses that children suffer from. Could you tell me more about those?**
  - Probe on how they are caused
  - Probe on those related to food or nutrition
  - Probe on their seriousness
  - Probe on ways to prevent them

- **I would like to hear about those illnesses of children that you are most concerned about.**
  - Probe on reasons why

Meal Preparation & Eating/Feeding Behavior

- **Let's talk about food now. Tell me about the food available to your community.**
  - Probe on seasonal foods
  - Probe on challenges related to food
  - Probe on special foods only consumed sometimes

- **I would like to hear more about special foods. Can you tell me more about special foods for pregnant women and young children?**
  - Probe on foods for and those not for young children under 2 years
  - Probe on foods for and those not for pregnant women
  - Probe on foods for and those not for pregnant women

- **Can you talk about what rules govern a traditional meal in this community?**
  - Probe on who prepares the meal
  - Probe on how a typical meal is cooked
  - Probe on how many people eat the meal
  - Probe on whether and how a family eats together

- **Could you explain the different kinds of meals that are prepared in your community?**
  - Probe on who decides what ingredients to get for preparing these meals
  - Probe on where she/he gets the ingredients to prepare these meals?

Developing a Child Nutrition Program

- **You're doing a great job. Now I want to hear some of your suggestions for developing a nutrition program that will introduce a food product such as this [hand MNP product to community leader and explain it to him/her] to children 6-23 months old in the community.**
- **Please tell me your impressions of this product.**
  - Probe on color, size, and other properties of the product packaging
  - Probe on any concerns about the product that are important to be aware of

- **Tell me some ways that you believe would be effective for promoting this product to caretakers in the community.**
  - Probe on ways that would be effective to promote the product
  - Probe on channels in the community to promote the product
  - Probe on preferred delivery channels of the MNP to the community
  - Probe on whether promotion should differ for male versus female caregivers
  - Probe on whether promotion should differ based on age of caregiver
  - Probe on other considerations related to promotion

- **Finally, I want to hear about some specific messages you believe would be effective in promoting this type of product**
  - Probe on how this product should be explained to caregivers

  - Probe on ways to ensure that it will be used appropriately
  - Probe on ways to promote its use but limit the sharing of it with other people in the household or community

- **Thank you for the information today. Do you have any additional questions or comments that could help us to develop a nutrition program with this type of product in your community?**

COMMUNITY LEADER INTERVIEW GUIDE
(HAUSA VERSION)

Gabatarwa

- **Ina godiya bisa damar da ka bani ta ganawa da kai a yau. Ina rokon da ka fara dan bincina min wani bayani akan karkararku.**
- **Zan so ka kwatanta min irin rawar da kake takawa a matsayinka na shugaban karkara.**
- **Ko zaka iya bayyana mana irin kalubalen da karkarar ku ta ke fuskanta?**
    - Bincike akan kalubalen da ke tattare da abinci

Ra'ayin Al'umma

- **Bayyana min tsarin da ku ka fi bi bisa rayuwa a wannan karkarar**
    - Bincike akan yan'uwantaka
    - Bincike akan addini
    - Bincike akan aikin kowa
    - Bincike akan tsarin shugabanci
    - Bincike akan masu maganin gargajiya a karakara
    - Bincike akan ma'anar zamantakewa cikin wannan Shirin

Cututtuka a karkara

- **Ka bayyana wasu daga cikin kalubale. Ko zaka iya gaya min cututtukan da ku ke fama da su a karkarar ku?**
    - Bincike akan abinda ke kawo su
    - Bincike akan karfinsu
    - Bincike akan hanyoyin kare kai

- **Yanzu za ka iya bayani akan ginuwar jiki ko cututtukan da ake kamuwa da su ta hanyar abinci a karkararku?**
    - Bincike akan ko rashin abinci mai gina jiki cutarwa ne
    - Bincike akan cutar da ake samu bisa rashin isashshen abinci

- **Na fi damuwa da irin cututtukan da suke addabar yara. Zaka yi min Karin bayani akan su?**
    - Bincike akan abinda yake haifar da su
    - Bincike akan nau'in abinci ko ginuwar jiki
    - Bincike akan karfinsu
    - Bincike akan hanyoyin kare kai

- **Zan bukaci jin bayanin cututtukan yara wanda ka fi damuwa da su.**
    - Bincike akan dalilanka

Hade-Haden abinci da/Tsarin ciyarwa

- **Mu tattauna akan abinci yanzu. Yi min bayani akan yalwatar abinci a karkararku.**
  - Bincike akan abincin yanayi
  - Bincike akan kalubalen da ke tattare da abinci
  - Bincike akan abinci na musamman da ake ci wasu lokuta

- **Zan so in ji bayani ga me da abinci na musamman. Zaka iya yi min Karin bayani akan abinci na musamman domin mace mai ciki da kananan yara?**
  - Bincike akan abincin da ya dace da wanda bai dace ba ga kananan yara yan'kasa da shekaru 2
  - Bincike akan abincin mata masu ciki da abincin da ba nasu ba
  - Bincike akan abincin mata masu ciki da abincin da ba na su ba

- **Zaka iya bayani akan tsarin da yake tafi da sha'anin abincin gargajiya a wannan karkarar?**
  - Bincike akan waye ya hada abincin
  - Bincike akan yadda aka dafa abincin
  - Bincike akan yawan mutanen da suka ci abincin
  - Bincike akan ta wace hanya iyalai ke haduwa su ci abinci tare

- **Zaka iya bayanin yawan nau'in abincin da ake hadawa a karkararku?**
  - Bincike akan waye yake yanke hukuncin wa'yanne irin sinadaran hada abinci yakamata ayi amfani da su wajen hada wannan abincin
  - Bincike akan inda ta/ya samo sinadaran hada wannan abincin?

Habbaka tsarin ginuwar jikin yara

- **Aikin ka yana kyau. Yanzu ina da bukatar in ji wasu daga cikin ra'ayinka domin habbaka tsarin ginuwar jiki wanda yake gabatar da kayan abinci kamar (masaniyar sinadari ga shugaban karkara na MNP)domin yara yan'watanni 6 – 23 da ke cikin karakara.**
- **Ina rokon ka bayyana min karbuwar wannan kayan a wajenka**
  - Bincike akan kala, girma, da kuma yanda madaukan kayan suke
  - Bincike akan hanyoyin da masu bada kulawa zasu karbi al'amarin
  - Bincike akan sauran matsalolin ga me da kayan

- **Zan so ka bayyana wasu hanyoyi da a tsammaninka zasu yi aiki wajen ciyar da wayennan kaya gaba ga masu bada kulawa a karkara.**
  - Bincike akan hanyoyin da zasu yi aiki wajen ciyar da kaya gaba
  - Bincike akan hanyoyin raba kayan a karkara
  - Bincike akan ko hanyar ciyar da kayan gaba zata banbanta a kan mata da maza masu bada kulawa
  - Bincike akan ko ciyar da lamarin gaba zai banbanta dalilin shekarun masu bada kulawar
  - Bincike akan sauran abubuwan da aka yarda da su dangane da ci gaba

- **Daga karshe, zan so in ji daga gareka ga me da sakonni na musanman wanda ka tabbata zasu taimaka wajen nasarar shigar da wannan kaya**
    - Bincike akan yanda za’a bayyana wa masu bada kulawa wannan kaya
    - Bincike akan hanyoyin da za’a yi aiki da wannan kaya
    - Bincike akan hanyoyin da za’a bayyana aikin wannan kaya amma a takaita rabawa

- **Muna godiya da bayanan da muka samu yau. Kana/kina da wata tambayar kuma ko Karin bayani wanda zasu taimaka mana mu habbaka tsarin ginuwar jiki da irin wannan kaya a karkararku?**
