# Supplementary material for: Acceptance and compliance with micronutrient powder (MNP) among children aged 6–23 months in northern Nigeria
Source: PLOS Glob Public Health. 2022 Oct 17;2(10):e0000961. doi: 10.1371/journal.pgph.0000961 (PMC10022258; doi:10.1371/journal.pgph.0000961)
Supplement: S2 File — (PDF) [file pgph.0000961.s002.pdf]

HEALTH WORKER INTERVIEW GUIDE
(ENGLISH VERSION)

Introduction

- **Would you tell me about your job in this community**
- **Tell me about your typical day from morning till evening here as a health care worker or volunteer**
  - Probe on tasks performed on the job

Illnesses in the community

- **Would you tell me the food related illnesses that children suffer from in this community?**
  - Probe on wasting, edema, stunting, and low blood levels (anemia)
  - Probe on how the health worker believes each illness is caused
  - Probe on how serious the health worker thinks each illness is
  - Probe on how susceptible young children are to each illness
  - Probe on how the health worker thinks each illness can be prevented
  - Probe on how the health worker thinks each illness can be treated

- **Which illnesses are you most concerned with?**
  - Probe on experience as a health care worker with a specific malnourished child patient
  - Probe for any specific stories/narratives

- **Would you talk about how caregivers understand each nutrition-related illness (wasting, edema, stunting, low blood levels (anemia), etc.) for children in this community?**
  - Probe on caregiver knowledge on how to prevent each of these illnesses
  - Probe on caregiver knowledge on what to do when a child has each illness
  - Probe on the common prevention measures that caregivers use for each illness
  - Probe on common treatment measures or ways that the caregivers use for each illness
  - Probe if there has been any awareness campaigns about the illnesses

- **Would you tell me about the follow ups on malnourished children in this community**
  - Probe on the advice they give to caregivers
  - Probe if the caregivers follow the advice
  - Probe on reasons why/why caregivers do not follow the advice
  - Probe on the treatments they give

- **Thank you for that great information about illnesses of children. I have one more set of illness questions, but related to women's health now. I am particularly interested in your experiences with female caregivers who have a loss of blood (anemia). o Could you tell me about your experiences with female caregivers who had anemia?**
  - Probe on the frequency of seeing these cases

- Probe on the severity of these cases
- Probe on the caregiver attitudes toward having this illness
- Probe on health worker perceptions of the causes of this illness in women of reproductive age
- Probe on how these perceptions differ between anemia for children and anemia for women of reproductive age
- Probe on health worker advice to women to prevent the illness
- Probe on health worker advice to treat this illness

<u>Food and feeding behaviors of children under two years</u>

- **Now I want to know about the foods that young children eat and their feeding behavior and habits in this community.**
  - Probe on the different types of food that children less than 2 years eat
  - Probe on foods that help prevent nutritional illnesses
  - Probe on if caregivers know about these foods and how they help
  - Probe on the availability of and access to these foods

<u>Developing a nutrition intervention</u>

- **Now I want to hear your suggestions about developing a nutrition program that's going to introduce a food product like this one (MNP) to children of 6-23 months**
- **What are your impressions of this product**
  - Probe on the color, size, packaging
  - Probe on how should this product should be modified to be accepted by caregivers
  - Probe on any concerns you have about the product
  - Probe on the taste
  - Probe if they see it as a food or medicine

- **Would you tell me about the effective ways of promoting this product in the community**
  - Probe on how the product should be distributed
  - Probe on other ways of promoting the product, including channels of message dissemination, forms of media, audience segments, etc.

- **Would you tell me the specific messages that would help in promoting the product or appeal to caregivers in this community.**
  - Probe on how this MNP should be explained to caregivers
  - Probe on ways to ensure that the product is being used appropriately
  - Probe on ways to make sure the product is being used appropriately
  - Probe on ways to limit sharing of this product among caregivers

- **Do you have any final thoughts or questions related to a program with this type of product?**

HEALTH WORKER INTERVIEW GUIDE
(HAUSA VERSION)

Gabatarwa

- **Shin zaka/zaki bayyana min aikin ka a wannan karkarar**
- **Yi min bayani kan yadda kowace rana ke kasancewa gareka a matsayinka na ma'aikacin lafiya ko mai aikin sa kai**
    - o Bincike akan yadda ake gabatar da aikin

Cututtuka a karkara

- **Zaka/zaki iya yi min bayani ga me da nau'in cututtukan da ya'yanku suka sha fama dasu ta hanyar abinci a wannan karkarar?**
    - o Bincike akan rama, kumburi, tsunburewa, karancin jini, da rashin ginuwar jiki
    - o Bincike akan hanyar da suke ganin ita ke haifar da cutar
    - o Bincike akan zaton da suke da shi ga me da karfin kowace dayan cutar
    - o Bincike akan hanyoyin da suke zaton za'a iya bi domin kariya daga kowace dayan cutar

- **Wace cutar ce kafi/kikafi damuwa da ita?**
    - o Bincike akan masaniyar da ka taba samu a matsayinka na ma'aikacin lafiya ga me da yaro maras lafiya wanda baya samun kulawa
    - o Bincike akan kowane irin labari na musamman/bayarwa

- **Zaka/zaki iya bayyana mana hanyoyin da masu bada kulawa suka fahimci nau'in abinci da cututtukan da ke tattare da shi a wannan karkarar?**
    - o Bincike akan ilimin yadda za'a sami kariya daga wayennan cututtuka
    - o Bincike akan masaniyar matakin da ya kamata a dauka yayin da yaro ya kasance baya samun kulawa
    - o Bincike akan matakan kariya wanda masu kulawa suke amfani da su
    - o Bincike akan matakan jinya ko hanyoyin da masu kulawa su ke amfani da su
    - o Bincike akan ko da akwai wayewar kai ga me da cututtukan

- **Shin zaka/zaki iya yi min bayani ga me da bibiya akan yaran da basa samun kulawa a wannan karkarar**
    - o Bincike akan shawarwarin da ake baiwa masu kulawa
    - o Bincike akan ko masu kulawa suna amfani da shawarwarin
    - o Bincike akan magantawar da suke yi

Dabi'un yara yan'kasa da shekaru biyu akan abinci da ciyarwa

- **Yanzu zan so a sanar da ni dabi'un kananan yara akan abincin da suke ci ko kuma yayin da ake ciyar da su a wannan karkara.**
    - o Bincike akan irin nau'ikan abincin da kananan yara yan'kasa da shekaru 2 suke ci
    - o Bincike akan nau'in abincin da ke taimakawa wajen kariya daga kamuwa da cuta

    - Bincike akan masaniyar da masu bada kulawa ke da ita ga me da wayennan abincin tare da yadda suke taimakawa
    - Bincike akan yawan abincin tare da saukin samun su

<u>**Habbaka hanyar samar da abinci**</u>

- **Zan so in ji irin tunaninka/ki ga me da hanyar habbaka tsarin samar da abinci wacce zata gabatar da tsarin (MNP) kamar wannan ga kananan yara yan'watanni 6 - 23**
- **Me zaka/zaki iya cewa ga me da wannan kaya**
    - Bincike akan kala, girma, madauki
    - Bincike akan ta wace hanya wannan kaya zasu karbu ga masu bada kulawa
    - Bincike akan ko wace irin damuwa/matsala ka ke da ita ga me da wannan kaya
    - Bincike akan dandano
    - Bincike akan ko su na ganin abinci ne ko magani

- **Shin zaka/zaki iya yi min bayani dangane da hanyoyin da suka fi dacewa abi domin tallata wannan kaya a karkara**
    - Bincike akan hanyoyin da suka dace abi domin raba wannan kaya
    - Bincike akan wasu hanyoyin na tallata kaya, hadi da sakon rashin inganci, fahimtar masu amfana, hanyoyin radio da kasidu, da sauransu

- **Shin ko zaka/zaki iya gaya min wasu sakonni da zasu iya taimakawa wajen tallata kaya ko lallashin masu bada kulawa a wannan karkarar.**
    - Bincike akan hanyar fahimtar da masu bada kulawa ga me da wannan abinci
    - Bincike akan tabbatar da bin hanyar da ta dace domin amfani da kaya
    - Bincike akan tabbatar da cewa ana amfani da kaya ta hanyar da ta dace
    - Bincike akan rage yadda masu bada kulawa suke karba-karba wajen amfani da kaya

- **Shin kana/kina da wani tunani na karshe ko tambaya ga me da wannan tsari akan irin wannan hoda?**
