# Supplementary material for: Acceptance and compliance with micronutrient powder (MNP) among children aged 6–23 months in northern Nigeria
Source: PLOS Glob Public Health. 2022 Oct 17;2(10):e0000961. doi: 10.1371/journal.pgph.0000961 (PMC10022258; doi:10.1371/journal.pgph.0000961)
Supplement: S3 File — (PDF) [file pgph.0000961.s003.pdf]

CAREGIVER INTERVIEW GUIDE
(ENGLISH VERSION)

Introduction

- **Thank you for speaking to me today. Please explain to me your typical day.**
- **Tell me more about your family please.**
- **Can you please discuss the challenges that you face here in your community?**

Illnesses in the Community

- **You mentioned some different challenges. Can you tell me more about the illnesses that your family has suffered from?**
  - Probe on how they are caused
  - Probe on their seriousness
  - Probe on ways to prevent them

- **Now could you talk about the nutrition or food-related illnesses in your household or community?**
  - Probe on whether micronutrient deficiency is an illness
  - Probe on whether being too short (stunting) is an illness

- **I am most interested in illnesses that children suffer from. Could you tell me more about those?**
  - Probe on how they are caused
  - Probe on those related to food or nutrition
  - Probe on their seriousness
  - Probe on ways to prevent them

- **I would like to hear about those illnesses of children that you are most concerned about.**
  - Probe on reasons why

Meal Preparation & Eating/Feeding Behavior

- **Let's talk about food now. Tell me about the food available to your family.**
  - Probe on seasonal foods
  - Probe on challenges related to food
  - Probe on special foods only consumed sometimes

- **I would like to hear more about special foods. Can you tell me more about special foods?**
  - Probe on foods for those not for young children under 2 years
  - Probe on foods for and those not for pregnant and lactating women

- **Can you talk about what a typical meal consists of in your household?**
    - Probe on who prepares the meal
    - Probe on how a typical meal is cooked
    - Probe on how many people eat the meal
    - Probe on whether a family eats together

- **Could you explain the range of meals that are prepared in your household?**
    - Probe on where she/he gets the ingredients to prepare these meals?

<u>Food Sharing</u>

- **Now I want to discuss the sharing of foods among people. Can you tell me more about how people share food in this community?**
    - Probe on sharing at the household level among family members
    - Probe on sharing at the community level among others
    - Probe on the stealing of food
    - Probe on reasons why sharing and stealing occur

- **Could you discuss preferences for some children over others with food allocation?**
    - Probe on gender preference (boys vs girls)
    - Probe on age preference (young vs older children)
    - Probe on quality and quantity of food preferences

- **Please talk to me about how young children under two years eat.**
    - Probe on how he/she determines what children eat everyday
    - Probe on food sharing that occurs among children
    - Probe on whether young children have decision-making capabilities in terms of food consumption.

- **Talk about any other child feeding/eating challenges that you face.**

<u>Developing a Child Nutrition Program</u>

- **You're doing a great job. Now I want to hear some of your suggestions for developing a nutrition program that will introduce a food product such as this [hand MNP product to caregiver] to children 6-23 months old in the community.**
- **Please tell me your impressions of this product.**
    - Probe on color, size, and other properties of the product packaging
    - Probe on ways it could be modified to be accepted by caregivers
    - Probe on other concerns about the product

- **Tell me some ways that you believe would be effective for promoting this product to caretakers in the community.**
    - Probe on ways that would be effective to promote the product
    - Probe on channels in the community to distribute the product
    - Probe on whether promotion should differ for male versus female caregivers

  - Probe on whether promotion should differ based on age of caregiver
  - Probe on other considerations related to promotion

- **Finally, I want to hear about some specific messages you believe would be effective in promoting the product.**
  - Probe on how this product should be explained to caregivers
  - Probe on ways to ensure that it will be used appropriately
  - Probe on ways to promote its use but limit the sharing of it

- **Thank you for the information today. Do you have any additional questions or comments that could help us to develop a nutrition program with this type of product in your community?**

CAREGIVER INTERVIEW GUIDE
(HAUSA VERSION)

Gabatarwa

- **Ina godiya bisa damar da ka/kika bani ta ganawa da kai/ke a yau. Zan so ka bayyana min ranar da kafi/kikafi so.**
- **Zan so in ji Karin bayani ga me da iyalanka/ki.**
- **Ko zamu iya tattauna irin kalubalen da kuke fuskanta a nan karkarar ta ku?**

Cututtuka a karkara

- **Ka/kin bayyana wasu daga cikin kalubale. Ko zaka kara bayyana min irin cututtukan da iyalanka/ki suka yi fama da su?**
  - Bincike akan abinda ke haifar da su
  - Bincike akan tabbatuwarsu
  - Bincike akan yadda za'a kare kai daga su

- **Yanzu ko zamu iya tattaunawa akan ginuwar jiki ko abincin da ya danganci cututtuka a gidajenku da karkararku?**
  - Bincike akan ko rashin abinci mai gina jiki yana cutarwa
  - Bincike akan ko karancin ginuwar jiki yana cutarwa

- **Na fi damuwa da irin cututtukan da yara su ka yi fama da su. Ko zaka/zaki yi min Karin bayani akan su?**
  - Bincike akan abinda yake haifar dasu
  - Bincike akan nau'in abinci ko ginuwar jiki
  - Bincike akan karfinsu
  - Bincike akan hanyoyin kare su

- **Zan so in ji Karin bayani akan wayennan cututtukan da ke addabar yara wanda kafi/kikafi damuwa da su.**
  - Bincike akan dalilai

Hada nau'in abinci da ci/Tsarin ciyarwa

- **Yanzu sai mu tattauna akan abinci. Ka/ki yi min bayani akan yawan nau'in abinci ga iyalanka/ki.**
  - Bincike akan abincin yanayi
  - Bincike akan kalubalen da ke kan abincin
  - Bincike akan abinci na musamman da ake ci wasu lokuta

- **Zan so in ji Karin bayani a kan abinci na musamman. Ko zaka/zaki iya yi min Karin bayani ga me da abinci na musamman?**
  - Bincike akan nau'in abincin da ba na yara yan'kasa da shekaru 2 ba ne

  - Bincike akan abincin mata masu ciki da masu shayarwa da kuma wanda ba nasu ba ne

- **Zaka/zaki iya bayani akan ko wane irin kayen hadi abinci na musamman yake kunsa a gidajenku?**
  - Bincike akan waye yake/take shirya abincin
  - Bincike akan ta yaya ake dafa abincin
  - Bincike akan ko mutum nawa ne suke cin abincin
  - Bincike akan ko iyalai suna cin abinci tare

- **Zaka/zaki iya yin bayanin yawan abincin da ake shiryawa a gidajenku?**
  - Bincike akan ko a ina ne ta/ya samo kayan cefane domin hada wannan abincin?

<u>**Ciyayya**</u>

- **Yanzu ina so mu tattauna akan yanda ake ciyayya a tsakanin al'umma. Ko zaka/zaki yi min Karin bayani akan ta yaya mutane suke ciyayya a wannan karkarar?**
  - Bincike akan ciyayya tsakanin magidanta cikin iyalai
  - Bincike akan yanda ake ciyayya a matakin karkara tare da mutane
  - Bincike akan satar abinci
  - Bincike akan dalilan da ke haifar da ciyayya da satar abinci

- **Zaka/zaki iya tattaunawa akan zabin da yara ke da shi wajen raba abinci?**
  - Bincike akan zaben jinsi (yara maza da mata)
  - Bincike akan zaben shekaru (yara kanana da masu yan'shekaru)
  - Bincike akan zaben inganci da yawan abinci

- **Ina rokon ka/ki yi min bayani akan yanda yara kanana yan'kasa da shekaru biyu ke cin abinci.**
  - Bincike akan ta yaya shi/ita ke iya tantance me da me yara ke ci kullum
  - Bincike akan yanda yara ke ciyayya a tsakanin su
  - Bincike akan ko kananan yara na iya yanke hukunci dangane da abinda zasu ci.

- **Kayi/kinyi bayani akan kalubalen da ka taba fuskanta wajen ciyar da yara.**

<u>**Habbaka abinci domin yara**</u>

- **Aikin ka/ki yana kyau. Yanzu zan so in ji wasu daga cikin ra'ayinka akan habbaka tsarin abinci wajen gabatar da kayan abinci kamar [masaniyar sinadari ga masu bada kulawa MNP}domin kananan yara yan'watanni 6 – 23 a karkara.**
- **Ina rokon ka/ki bayyana min tunaninka akan wannan kayan.**
  - Bincike akan kala, girma, da madaukin kayan
  - Bincike akan hanyoyin da za'a bi domin saukaka aikin masu badakulawa
  - Bincike akan sauran matsalolin da za'a iya fuskanta ga me da kayan

- **Yi min bayani akan hanyoyin da kake/kike ganin zasu iya taimakawa wajen kwarzanta wannan kaya ga masu bada kulawa a karkara.**
  - Bincike akan hanyoyin da ka iya kwarzanta kaya
  - Bincike akan hanyoyin da za’a bi domin raba kaya
  - Bincike akan ko tallata kaya zai banbanta a tsakanin maza da mata masu bada kulawa
  - Bincike akan ko tallata kaya zai banbanta ta hanyar shekarun masu bada kulawa
  - Bincike akan sauran tsari domin tallata kaya

- **Daga karshe, zan so in ji ko kana/kina da wasu sakonni da kake da yakinin zasu taimaka taimaka wajen tallata kayan.**
  - Bincike akan ta yaya za’a yiwa masu bada kulawa bayanin wannan kaya
  - Bincike akan hanyoyin da za’a tabbatar anyi amfani da su
  - Bincike akan hanyoyin tallata amfanin kaya da rage Hadaka wajen amfani da su

- **Muna godiya da bayanan da muka samu yau. Kana/kina da wani Karin bayani ko tambaya wanda zasu iya taimaka mana wajen habbaka tsarin abinci da irin wannan kaya a karkararku?**
