# Supplementary material for: Acceptance and compliance with micronutrient powder (MNP) among children aged 6–23 months in northern Nigeria
Source: PLOS Glob Public Health. 2022 Oct 17;2(10):e0000961. doi: 10.1371/journal.pgph.0000961 (PMC10022258; doi:10.1371/journal.pgph.0000961)
Supplement: S4 File — (PDF) [file pgph.0000961.s004.pdf]

MixMe Messages for Caregivers

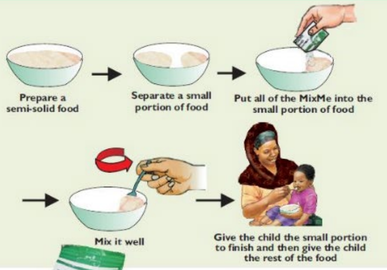

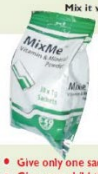

MixMe is a mixture of vitamins and minerals to improve your child's health and nutrition

- Give only one sachet of MixMe per child 6 - 23 months per day.
- Give your child this MixMe for up to 60 days.
- If you missed giving MixMe to your child, then you should still continue giving him/her one sachet everyday until it is finished.

Remember:
- Do not add MixMe to food during cooking
- Do not add MixMe to water or other liquids

for further inquiries please contact your nearest health clinic

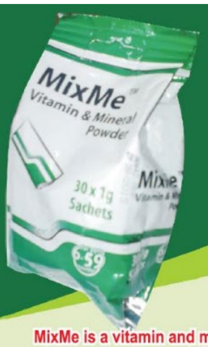

MESSAGES FOR CAREGIVERS ON MixMe

MixMe is a vitamin and mineral powder which makes food nutritious. Children become stronger and more intelligent

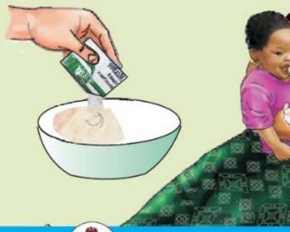

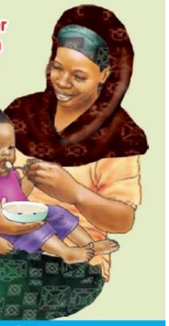

unicef

INFANT AND YOUNG CHILD NUTRITION

* In Nigeria, 4 out of 10 children suffer from malnutrition
* Anaemia (lack of blood) is a problem in Nigeria affecting up to 7 out of 10 children
* Most of the brain development occurs between birth and 2 years of age
* A nutritious diet is essential for the proper growth and development of the brain
* The food we eat may not contain enough nutrients and vitamins to support the growth and development of the body

RECOMMENDED INFANT AND YOUNG CHILD FEEDING

* Newborn should be put to mother's breast within 30minutes of delivery
* The mother's milk contains all the required nutrients a child needs and so they should be given breast milk only from birth till 6 months. Infants should not be given water before 6 months.
* From 6 months, children should be fed with complementary food in addition to breast milk as breast milk alone is no longer sufficient

WHAT IS MICRONUTRIENT POWDER

Micronutrient Powder is a mixture of 15 types of vitamins and minerals including iron. It is a small sachet and should be fed by being mixed into a semi-solid food such as pap, kunu or other gruel. MNP improves nutrient quality oflocally available foods, making it more nutritious for your child thus ensuring optimum growth development of the child.

WHAT ARE THE BENEFITS OF USING MNP

* It makes the food more nutritious for the child
* MNP makes children healthier, more intelligent and stronger
* Children who are fed with MNP are less likely to lack blood (anaemia)
* MNP may increase child's appetite

WHO TO GIVE MICRONUTRIENT POWDER

* Children between 6 – 23 months, because this is the age when brain development is highest and damage will be irreversible

WHO NOT TO GIVE MICRONUTRIENT POWDER

* Children under 6 months
* Children receiving treatment for Severe Acute Malnutrition. MNP can be given after completion of the treatment
* Children suffering from malaria. MNP can be given after treatment

HOW TO GIVE MICRONUTRIENT POWDER

* MNP should only be given to children above 6 months as before then, breast milk only is adequate for the child
* Only 1 sachet of MNP should be fed to 1 child every day for 60 days
* MNP should be mixed into a small portion of semi-solid food and fed to the child within half an hour of mixing
* MNP should not be mixed with very hot food or liquids
* If you forget to give child MNP, continue using MNP as soon as you remember
