# Supplementary material for: Acceptance and compliance with micronutrient powder (MNP) among children aged 6–23 months in northern Nigeria
Source: PLOS Glob Public Health. 2022 Oct 17;2(10):e0000961. doi: 10.1371/journal.pgph.0000961 (PMC10022258; doi:10.1371/journal.pgph.0000961)
Supplement: S5 File — (PDF) [file pgph.0000961.s005.pdf]

FULL-DAY DIRECT OBSERVATION FORM

Date: ______________________Location: __________________________________

Data Collector: ______________________Participant ID: ________________________

Main goals of this direct observation are to answer the following questions:<br><br>1. In this household, what would be the best way(s) to give nutritional supplements to young children? Why?<br><br>2. In this household, describe the food sharing that you observe throughout the meal. Are there predictable patterns?<br><br>3. In this household, who is favored and how is this favoritism expressed during meal preparation and eating?

Household Composition Table (Fill out as you do data collection)

INITIALS	SEX (M/F)	AGE (estimated)	Relationship to household	Description

PARTICIPANT ID: _________

TIME	ACTOR INITIALS	RECIPIENT INITIALS	WHAT HAPPENED	FOOD	Reactivity

TIME	ACTOR INITIALS	RECIPIENT INITIALS	WHAT HAPPENED	FOOD	Reactivity

TIME	ACTOR INITIALS	RECIPIENT INITIALS	WHAT HAPPENED	FOOD	Reactivity

TIME	ACTOR INITIALS	RECIPIENT INITIALS	WHAT HAPPENED	FOOD	Reactivity

Direct observation field notes (Complete after you complete data collection):

From what you have seen observing this household, please answer the following questions in detail:

Field notes question	Write your detailed response here
1. In this household, what would be the best way(s) to give nutritional supplements such as an MNP to young children to ensure compliance? Why?	
2. In this household, who is favored during meal preparation or feeding? How is this favoritism expressed?	
3. How could we promote the MNP supplement for this family that would attract their attention?	
4. In this household, what would be the best ways to promote the MNP but limit how much it may be shared? Why?	
5. What other issues and concerns did you see with this family that are important for us to consider with regard to introduction of an MNP? Why?	
6. Based on your experience observing this child today, do you believe reactivity negatively impacted your ability to see the food-related behaviors of the child?<br><br>Please explain your answer in detail.	
