# Supplementary material for: Acceptance and compliance with micronutrient powder (MNP) among children aged 6–23 months in northern Nigeria
Source: PLOS Glob Public Health. 2022 Oct 17;2(10):e0000961. doi: 10.1371/journal.pgph.0000961 (PMC10022258; doi:10.1371/journal.pgph.0000961)
Supplement: S6 File — (PDF) [file pgph.0000961.s006.pdf]

MNP INTERVIEW GUIDE (FOR CAREGIVERS)
(ENGLISH VERSION)

Introduction

- **Describe for me the people in your family.**
    - Probe on specific gender and age of the child 6-23 months (if caregiver)

Young children 6-23 months

- **Explain to me your young child's 6-23 months day from when he/she wakes up until bed**
- **We have heard of food challenges for young children in this setting related to the seasons**
    - Probe on coping mechanisms to these food challenges
    - Probe on how challenges affect young child feeding and eating habits
    - Probe on how they affect the young child's eating behaviors

Experience with MNP

- **Now I want us to talk about the MNP that you received. Could you tell me about your experience with the product?**
    - Probe on positive aspects of the experience, especially for stories/anecdotes
    - Probe on challenges with the experience, especially for stories/anecdotes
    - Probe on barriers that made it hard to use it appropriately everyday (structural, psychosocial, etc…)

- **Some mothers indicated that it might be hard to remember to use every day. Did you have a similar experience and what did you do to remember?**
    - Probe on solutions that the caretaker suggests to address each abovementioned barrier
    - Probe on facilitating factors that made it easier to use everyday
    - Probe on feeding methods using the product including foods with which it was mixed

- **Let's talk about the child's response to the MNP**
    - Probe on the extent that the child likes or dislikes the taste of the product
    - Probe on how, if at all, consumption of MNP impacted the child's current diet

- **In other villages we noticed that sharing/stealing of the product occurred. Could you talk about sharing/stealing of the product when you used it?**
    - Probe on sharing with other children in household or community
    - Probe on sharing due to food shortage during lean season
    - Probe on theft of the product by other community members
    - Probe on caretaker or father eating the product as a snack

- Probe on leftovers of the product being consumed by others
  - Probe on whether 28 sachets were enough for first 28 days. Why/Why not?

- **Now that you have used the product, could you talk about how you perceive it?**
  - Probe on how the caretaker perceives the product (e.g., medicine versus food)
  - Probe on perceived benefits of this product?
  - Probe on whether this person would purchase this product if it were cheaply on sale in her village
  - Probe on how much she would be willing to pay for each sachet

- **You are giving me great information. Now please tell me some ways that you believe would be effective for promoting this product to caretakers in the community.**
  - Probe on ways that would be effective to promote the product for its appropriate use
  - Probe on channels in the community to effectively distribute the product
  - Probe on whether promotion should differ based on characteristic of caretaker
    - Males versus females
    - Age of caretaker
    - Social status of caretaker
    - Number of children of caretaker

- **I want to hear some specific messages you think would effectively promote the product.**
  - Probe on what messages should be used to explain this product to caretakers.
  - Probe on specific messages to ensure that it will be used appropriately.
  - Probe on effective messages to promote its use but limit the sharing of it
  - Probe on any messages that would be useful to convey on the sachet itself

- **Finally, is there anything else that you can share about your experience with this product?**
  - Probe on anything else about the product, placement, price, promotion
  - Probe on anything else the caregiver or community leader can recommend to develop an effective program

- **Thank you for that information today. Do you have any additional comments or questions to help inform our nutrition program with this type of product?**

MNP INTERVIEW GUIDE (FOR CAREGIVERS)
(HAUSA VERSION)

Gabatarwa

- **Fasalta min mutane dake tattare a iyalanku.**
    - Bincike akan ainihin jinsi da watannin yara tsakanin wata 6-23 (wanda ake baiwa kulawa)

Kananan yara yan'watanni 6-23

- **Bayyana min wata rana guda wacce dan'ki/yar'ki yan'wata 6-23 in sun farka daga barci basa sake komawa sai lokacin wani barcin (wanda ake baiwa kulawa)**
- **Mun ji irin kalubalen da ake fama da shi wajen abincin kananan yara cikin wannan tsarin na yanayi**
    - Bincike akan kula da sinadarai domin wayennan kalubalen abincin
    - Bincike akan yanda kalubale ke shafar karamin yaro wajen Halaiyar cin abinci
    - Bincike akan yanda kalubale ke shafar dabi'un kananan yara wajen cin abinci

Fahimtar hoda mai sinadaran gina jiki

- **Yanzu ina son mu tattauna akan hoda mai sinadaran gina jiki wacce ki ka karba. Zaki iya yi min bayanin fahimtar da ki ka yi mata?**
    - Bincike akan karfafa tunanin fahimta, musamman wajen bada labarai/masu ban dariya
    - Bincike akan kalubalen da ke cikin fahimta, musamman wajen bada labarai/masu ban dariya
    - Bincike akan tarnakin da ke kawo tunanin shan wahala wajen amfani da hodar yanda ya kamata a kullum (cikin tsari, cikin wayewar kai, da sauransu…)
    - Wasu iyaye mata suna fadin cewa zai yi matukar wuya a dinga tunawa da yin amfani da ita a kullum. Kina da irin wannan fahimtar kuma me ki ke yi domin tunawar?
    - Bincike akan hanyoyin da mai bada kulawa ya shawartar domin abi don shawo kan tarnakin da aka bayyana a sama
    - Bincike akan hanyoyin koyarwar da suke bayyana saukin amfani da ita a kullum
    - Bincike akan tsarin ciyarwa tare da amfani da abincin da aka hada da ita

- **Yakamata mu tattauna akan amsuwar amfani da MNP ga yara**
    - Bincike akan karfin kauna ko rashin kaunar da yara suke yiwa dandanon hodar
    - Bincike akan ta yaya, in har zai yiwu, sha ko cin MNP zai sami tasiri cikin abincin yara

- **A wasu kauyukan mun fahimci cewa ciyayya/satar kayan yana wanzuwa. Ko zaki/kayi bayani akan ciyayyar/satar kayan bayan amfani dasu?**
    - Bincike akan ciyayya tare da sauran yara a cikin gida ko karakara

- Bincike akan ciyayya bisa dalilin karancin abinci a lokacin bukata
    - Bincike akan yanda wasu daga cikin mutanen karkara ke satar kayan
    - Bincike akan yanda uba ko mai bada kulawa ke cin kayan a matsayin taba ka lashe
    - Bincike akan yanda wasu ke cinya abinda yayi saura daga cikin kayan
    - Bincike akan ko fakiti 28 zasu isa har tsawon kwana 28. Abinda yasa/me yasa ba zasu isa ba?
    - To yanzu tunda kin/ka yi amfani da ita, ko zaka/ki yi bayani akan ta yaya za'a yi amfani da ita? (misali, hadin abinci da magani)
    - Bincike akan samun amfanin ta?
    - Bincike akan ko wannan matar zata sayi wannan hoda idan ta kasance mai saukin kudi a kauyensu
    - Bincike akan shin nawa zata iya sayen kowane fakiti

- **Kina bani bayyanai masu gamsarwa. Yanzu zan so ki bayyana min wasu hanyoyin da a fahimtar ki zasu kasance masu amfani wajen tallata wannan hoda ga masu bada kulawa a karkara.**
    - Bincike akan hanyoyin da zasu kasance masu amfani wajen tallata hodar tare da amfaninta
    - Bincike akan hanyoyin da suka kamata abi domin rarraba wannan hoda a karkara
    - Bincike akan ko tallatawar zata kasance ta hanyar matsayin masu bada kulawa
        - Maza tare da mata
        - Shekarun masu bada kulawa
        - Matsayi/wayewar masu bada kulawa
        - Yawan ya'yan masu bada kulawa

- **Zan so in ji wasu sakonni na musamman da kike zaton zasu kasance masu amfani wajen tallata hodar.**
    - Bincike akan wane irin sako yakamata ayi amfani da shi wajen bayanin wannan hoda ga masu bada kulawa.
    - Bincike akan sako na musamman domin tabbatar da amfani da ita ta hanyar da ta dace.
    - Bincike akan sako mafi inganci wajen tallata amfaninta amma tare da takaita rarraba ta
    - Bincike akan kowane irin sako wanda zai kasance mai amfani wajen rarraba ta cikin fakitin nata

- **Daga karshe, ko akwai wani Karin bayani da zaki karar kuma mu amfana a tsakaninmu ga me da fahimtar ki da wannan hoda?**
    - Bincike akan ko ma menene akan hodar, inda yakamata a saka ta, farashi, tallatawa
    - Bincike akan ko ma menene wanda mai bada kulawa ko shugaban karkara zasu yarda ka bada dama domin a yi kyakkyawan shiri kuma mai amfani

- **Muna godiya da wayennan bayanai na yau. Kina/kana da wani Karin bayani na musamman ko tambaya wacce zata taimaka mana akan shirinmu na tsarin ciyarwa tare da wannan hoda?**
